# Improving hand hygiene: thinking outside the bottle

**DOI:** 10.1186/1753-6561-5-S6-P116

**Published:** 2011-06-29

**Authors:** D Harkin, M-L McLaws

**Affiliations:** 1 School of Public Health and Community Medicine, University of New South Wales, Kensington, Australia

## Introduction / objectives

Hand hygiene is an integral aspect of infection control. The provision of Alcohol Based Hand Rub (ABHR) gel at patient bedsides has made effective hand hygiene (HH) more convenient for Health Care Workers (HCWs) by overcoming two of the main ‘barriers’ to HH- time and distance to sinks, by placing the bottles at the point of patient care. The ABHR bottles, being novel, ubiquitous and usually brightly coloured, were originally very effective in cuing HCWs to observe HH. In the years since their introduction, these attributes have not translated into HH rates much above 60%. This suggests that AHR bottles may not have retained the cue to memory they originally held.

## Methods

A series of direct observations and interviews with HCWs have suggested that the ABHRbottles, in the modern, busy patient environment are not as noticeable as they once were. Even if they are brightly coloured bottles, HCWs have become inured to their presence. We drew upon the practice of commercial manufacturers of similar products- like liquid soap and shampoo. They constantly modify and refresh the external attributes of their products to maintain their profile in the public eye. We developed a study to test whether such an approach could have the same effect in a clinical setting. We developed a study which involves modifying the external attributes of the ABHR bottles to refresh them in the eyes of HCWs- and test whether they can improve HH rates as a result of these modifications.In addition to measuring HH compliance rates, it will have a qualitative aspesct to gauge the perceptions of HCWs taking part in the study.

## Results

The study will begin at a Sydney teaching hospital in May 2011.

## Conclusion

This study will determine whether this novel approach can assist and support HCWs in complying with HH protocols.

## Disclosure of interest

None declared.

